# How and why we should move beyond natural selection in museums to tackle teleology

**DOI:** 10.1186/s12052-023-00184-8

**Published:** 2023-03-17

**Authors:** Shelley L. Smith

**Affiliations:** Department of Sociology and Anthropology, University of Texas at Arlington, Box 19599, Arlington, TX 76019 USA

**Keywords:** Evolutionary forces, Evolutionary mechanisms, Mutation, Genetic drift, Gene flow, Stochastic

## Abstract

**Background:**

Museum displays commonly use a “VIST” approach (Variation, Inheritance, Selection, and Time) to explain evolution to visitors. I contend that this framework, by focusing narrowly on natural selection, unintentionally reinforces intuitive teleological thinking and a “survival of the fittest” mentality. Exhibits that incorporate all the forces (or mechanisms) of evolution will instead challenge visitors’ preconceptions and enable them to develop a deeper understanding of evolution. In particular, visitors will appreciate that evolution is not progressive, with modern humans as the “most evolved” species.

**Results:**

Explicit and implicit description of the forces of evolution is surveyed in 12 museums: 4 in Texas, 7 elsewhere in the U.S., and the Natural History Museum in London. Museum exhibits focus primarily on natural selection (explicit in 10 of 12) and often mention mutation (explicit in 7). Only the American Museum of Natural History in New York, in my sample, provides an explicit explanation of genetic drift.

**Conclusions:**

Heavy emphasis on natural selection and limited explanation of stochastic forces contributes to an impoverished view of evolution. Exhibits should more effectively convey the complexity of microevolution. Computer simulations showing the interactions of evolutionary forces can accomplish this goal.

## Background

As part of a project exploring human evolution exhibits in museums, in 2016–2017 I examined the *Explore Evolution* exhibit that formerly was on display at the Texas Memorial Museum. In this exhibit, I learned that museum educators use a “VIST” framework to explain evolution to museum visitors. Variation, Inheritance, Selection, and Time were listed here as the *forces* of evolution. Having taught human evolution for decades, this came as a surprise to me, because what “VIST” explains is *one* of the forces (or mechanisms) of evolution, namely natural selection.^1^ As I subsequently discovered, science and natural history museums focus heavily on natural selection, often omitting other forces or mentioning them only in passing. The goal of this article is to indicate how rarely museums provide explicit content on evolutionary forces beyond natural selection, why this state of affairs is problematic, and how evolution exhibits can be improved.

I contend that focusing heavily only on natural selection leaves museum visitors with an impoverished view of evolution and does little to address some common misconceptions. In particular, I here target teleology, the idea that evolution is goal-directed. (See the special issue on Teleology and Evolution Education in this journal for extensive discussion, including important distinctions among types of teleological arguments; Hammann and Nehm 2020). While visitors intuitively understand adaptation and thus may grasp the basic idea of how natural selection works (but see Gregory 2009), leaving out “random” (better, stochastic) factors reinforces mistaken popular assumptions. Leaving a teleological mindset intact is problematic, I submit, because visitors continue to view evolution as necessarily progressive, with modern humans at the pinnacle. A “survival of the fittest” mentality prevails, in the Spencerian competitive sense rather than the Darwinian one of reproductive fitness. It is easy, and comfortable, to view ourselves as the “most evolved.”

Dan Wormald, a Learning Researcher in the Department of Learning Research and Evaluation at London’s Natural History Museum, commented in my 2017 interview with him that he thought “it would be perfectly possible to go through that [human evolution] exhibition and come out of it still with firmly fixed, teleological ideas about evolution, so I’d like to be more up-front about the theoretical basis that that’s built on.” He elaborated with specific discussion of Deep Time and our reluctance to admit we are animals, but the same point holds with regard to the forces of evolution. An internal Summative Evaluation Report for the human evolution gallery that Wormald kindly provided to me reinforces the concern that visitors leave without understanding how evolution works: “Whilst the exhibition is successfully conveying the non-linear nature of human evolution, the evaluations provide very little evidence that the exhibition is deepening visitors’ understanding of how evolution operates and of the mechanisms that drive the changes seen in the fossil record” (Wormald 2016).

Ideally, museum exhibits would facilitate this richer comprehension, but accomplishing that goal is not easy because it requires a cognitive shift or significant conceptual changes. Teleological reasoning is part of a natural and expected sequence in cognitive development. Spiegal et al. (2012) tested visitor understanding before and after viewing an installation of *Explore Evolution* and found improvements in evolutionary reasoning following engagement with the exhibit, but also increases in need-based explanations as opposed to desire-based ones, which the authors propose to be an intermediate step forward. However, as these authors recognize, both desire-based and need-based explanations are essentialist and focus on individuals rather than populations; both ignore pre-existing variation within *populations*. Importantly, no visitor surveyed in their study strongly agreed only with the evolutionary reasoning explanations while dismissing all intuitive and creationist options. Instead, all visitors exhibited mixed-reasoning patterns. Even college students’ understanding of natural selection can be impaired by teleological reasoning (Barnes et al. 2017). A comparatively small percentage of people have a sophisticated understanding of evolution. Teleology has a “long shadow” (Werth and Allchin 2020).

Batzli et al. (2016) propose variation as a threshold concept in biology and evolution. (For an introduction to threshold concepts, see Meyer and Land 2003). The developers of the Darwin 2009 museum project at the Zoological Museum of Rome similarly used framing concepts, two of which involved diversity at a variety of levels, to create exhibits and experiences facilitating constructivist knowledge opportunities for visitors (Falchetti 2012). A museum setting is ideal for such activities. Appreciating inter-individual variation led Darwin to propose his mechanism of natural selection, yet even today, crossing the threshold from typology and essentialism to an appreciation of variation may be difficult for many, especially if they associate variation with deviation from “normal.” (For examples of practical medical consequences of such an interpretation, see Trevathan 2007). Garvin-Doxas and Klymkowsky (2008) found that students often equated natural selection and evolution and displayed an “anti-random” bias, thinking of “random” processes as inefficient. Although VIST literally starts with Variation, the key significance of this may be missed by museum visitors. In attempting to hammer home how natural selection works, we may be missing an opportunity to promote a more expansive view of evolution that directly challenges misconceptions. We need to explain *where* variation comes from and *how* that variation is affected by all the forces of evolution.

## Methods

The data for this study derive from a larger survey of human evolution exhibits in museums. For the primary study, four museums were selected in Texas, seven elsewhere in the U.S., and one beyond the U.S. (the Natural History Museum in London, which serves as an “outgroup” for comparison with U.S. museums). All museums other than the Fort Worth Museum of Science and History (a comparison or control site with no human evolution content) were selected based on their human evolution exhibits and geographic locations, representing a range of museum experiences. The one currently most distinct is the Museum of Us, formerly known as the San Diego Museum of Man. Prior to its renaming, this museum contained a dated (installed 2002) but extensive human evolution exhibit. This sole Anthropology museum in my sample has in recent years moved increasingly toward cultural anthropology exhibits and programming. The former evolution exhibit, *Footsteps Through Time*, and a related section on human biology have been removed, but the *Race, Are We So Different?* exhibit created by the American Anthropological Association and the Science Museum of Minnesota has been retained (museumofus.org/exhibits/race-are-we-so-different 2022). I collected data on the former exhibits in 2016 and on the *Race* exhibit in 2016 and 2018. While my research on evolution is human-focused, arguably museum visitors can learn evolutionary principles effectively in this context due to its direct relevance to their lives (see Discussion).

Although my main focus in my primary research project was human evolution exhibits, I collected data from other evolution-focused exhibits throughout the museums to gain broader context for the targeted human evolution exhibits. Data collection was qualitative, utilizing notes and photographs of exhibits. For the secondary study reported here, I reviewed the coverage of natural selection, mutation, genetic drift, and gene flow—the four standard forces or mechanisms of evolution—in the 12 museums sampled. I have extensive documentation of the human evolution exhibits in these museums and substantial but less detailed information from the entirety of evolution-related exhibits throughout these museums. I acknowledge that in this secondary, retrospective study, I may have missed mention of one or more of the forces of evolution somewhere in the museums surveyed, and possibly even within the human evolution sections. The retrospective nature of this survey is an acknowledged limitation of this study. However, given that I visited these museums as a researcher specifically focused on evolution, I maintain that if I missed mention of these forces, the majority of visitors would likewise.

Box 1: examples of E and I codes

**Table:** 

	E = Explicit	I = Implicit
	National MNH	National MNH
Natural Selection	Evolution Trail, Hall of Mammals	Hall of Human Origins
	“Look around you. Like the members of all other species, every human has individual variations. Certain variations may help an individual survive and reproduce, especially if the environment is changing. Through natural selection these beneficial variations accumulate over many generations and spread through the population, becoming what biologists call adaptations.”	“To survive, living things adapt to their surroundings. Occasionally a genetic variation gives one member of a species an edge. That individual passes the beneficial gene to its descendants. More individuals with the beneficial trait survive and pass it to their descendants. If many beneficial traits arise over time, a new species – better adapted to its environment – evolves.”
Mutation	Texas MM, *Explore Evolution*	San Diego MM, *Race, Are we so different?*
	“If our DNA is so similar, why do we look so different? Scientists have discovered that tiny changes in DNA can cause big changes in bodies. For example, some DNA sequences control many other genes, turning them on or off. If one of these master control sequences mutates, it can alter the way all the other genes act. Scientists suspect that mutations in master control sequences may be one reason why humans and chimpanzees look so different.”	“Genetic variation refers to the natural differences in DNA sequences found in a population. These differences exist because tiny, random changes are always occurring in DNA and accumulate over time. Almost all of these differences are ‘silent’ and don’t affect us in any way. But some are at the root of each person’s unique appearance” (Note: E code in this exhibit for sickle cell anemia content.)
Genetic Drift	American MNH, Hall of Human Origins	Cal Acad Sci, *Human Odyssey*
	“Chance Takes Over. When a trait is completely neutral—neither helpful nor harmful—an unpredictable process called drift causes random changes in the frequency of a trait over many generations.” (Butterfly wing spots example follows.)	Lights display of a genetic bottleneck. (See weblink, California Academy of Sciences 2022, in references.)
Gene Flow	None recorded (in context of evolutionary forces)	London NHM, Human Evolution Gallery
		Discussion in video presentation of interbreeding among Neanderthals and *Homo sapiens* migrants

Coverage of evolutionary forces/mechanisms is coded as “E” for explicit or “I” for implicit. An “E” indicates explicit mention using the name (e.g., drift or genetic drift). An “I” indicates that the textual discussion does not name the force/mechanism or provide an explanation of it. However, visual and/or textual content is present that aids conceptual or intuitive understanding of its operation (e.g., a representation of a genetic bottleneck). See Box1 for examples of E and I codes. Importantly, I do not include “adaptation” within an implicit discussion of natural selection. In my experience, many people interpret adaptation as acclimatization (i.e., physiological changes obtained during an individual’s lifetime) or, similarly, as anatomical or morphological changes that can be acquired within one generation by individuals (see also the Adaptation section in Gregory 2009). Even if the exhibit text is meant to be interpreted as adaptation via natural selection, a visitor might not infer the intended meaning from the word “adaptation” alone. Several exhibits discuss migration; I chose not to code this as coverage of gene flow, even implicitly, unless interbreeding was put in the context of evolutionary forces (and specifically microevolution).

An I code is not necessarily inferior to an E code. For example, the mere mention of natural selection, with little to no explanation, does not give visitors an understanding of the operation of natural selection. However, explicit use of scientific terminology provides visitors with the words necessary to conduct further investigation on their own. It thus empowers visitors’ self-explorations of scientific concepts.^2^

## Results

A summary of the results appears in Table 1.

**Table 1:** Explicit (E) or Implicit (I) Coverage of Evolutionary Mechanisms

Museums	CODES^a^			
	Natural selection	Mutation	Genetric drift	Gene flow
Ft. Worth MSH				
Perot MNS	E	E	I	
Texas MM	E	E		
Houston MNS	E			
Denver MNS	E	E		I
Field Museum	E			
Cleveland MNH			I	
Cal Acad Sci	E	E	I	
San Diego MM	E	E		
National MNH	E	E		
American MNH	E	E	E	
London NHM	E			I

^a^E = name of mechanism mentioned; I = text does not name the mechanism or provide an explanation of it, but visual or textual content aids understanding of its operation. See Box1 for examples

### The Fort Worth Museum of Science and History (Fort Worth, Texas); Coding: No E’s nor I’s

Although geologic time periods (implying Deep Time) are present, I found no explicit or implicit discussion of the forces/mechanisms of evolution in this museum.

### The Perot Museum of Nature and Science (Dallas, Texas); Coding: Natural Selection, E; Mutation, E; Genetic Drift, I

The Genetic Foundations of Evolution section in the Discovering Life Hall defines mutation; How Evolution Happens describes natural selection using the VIST framework. Gene flow is not discussed, but the hall contains an illustration of a genetic bottleneck, and Sewall Wright is among those featured for his contributions to evolutionary theory. The Being Human Hall contains a Variation and Migration computer video.

One especially good physical interactive in the Discovering Life Hall is the Genetic Lottery Station. Here, two dragons, a mom and dad, have phenotypically different horns and tails but the same type of wings (genotype, mom, aaBbDD; genotype, dad, AaBbdd). Rotating blocks show various offspring outcomes for genotypes and phenotypes, indicating how new variation can arise. An “adapter reactor” kids’ game containing cartoon figures is used to show fanciful morphological changes due to natural selection. Similar ones showing more human-like figures appear in Cleveland and (formerly) at the Smithsonian. The latter was removed due to misinterpretation by visitors (see Discussion).

### The Texas Memorial Museum (Austin, Texas, 2016–2017); Coding: Natural Selection, E; Mutation, E

This museum formerly displayed *Explore Evolution*; in Austin, a comparison of chimpanzee and human skeletons was added to the baseline content of this exhibit. Funded by the National Science Foundation and developed in a collaborative partnership between the University of Nebraska State Museum and the Science Museum of Minnesota, this exhibit was formerly displayed in several university museums and remains on display in Nebraska (explore-evolution.unl.edu 2022). Mutation is mentioned in *Explore Evolution* in the context of “master control sequences” in DNA and for date estimation (“molecular clock”). Natural selection is discussed as part of the VIST framework.

### The Houston Museum of Natural Science (Houston, Texas); Coding: Natural Selection, E

Within the Morian Hall of Paleontology, there is no specific introduction to the forces of evolution and how they operate beyond a brief reference to natural selection. (Note that in Texas, with the removal of *Explore Evolution* in Austin, the Perot is the sole museum in my sample with such coverage.) A human evolution section is part of the “Prehistoric Safari.” This hall focuses on the fossil evidence for evolution. For more specifically on human evolution in Texas museums, see Smith (2020).

### The Denver Museum of Nature and Science (Denver, Colorado); Coding: Natural Selection, E; Mutation, E; Gene Flow, I

In the Genetics of Taste Lab within *Expedition Health*, visitors can contribute their DNA for research—you can become part of the museum’s collection!—but the genetics focus for the public is not on evolution. Within *Prehistoric Journey*, the museum’s walk thorough geologic time, the majority of the forces of evolution are covered. In a natural selection game, you become an insect predator. Mutation appears in the context of bacterial reproduction, with the clarifying example of the evolution of antibiotic resistance. The peppered moth example includes mutation and natural selection. Interbreeding (but not gene flow explicitly) is mentioned along with the gene pool concept in this section. In addition to the forces covered, *Prehistoric Journey* contains excellent graphics and exhibits for conceptualizing Deep Time, relative dating, and absolute dating. Senior Exhibit Developer Frances Kruger, who was a key member of the team for *Prehistoric Journey*, also consulted on the Discovering Life Hall for the Perot (personal communication). Her input thus helped both museums present explanations and interactives explaining evolutionary concepts.

### The Field Museum (Chicago, Illinois); Coding: Natural Selection, E

In most ways the *Evolving Planet* exhibit at the Field Museum is excellent, including its “Evolution: How it works and how we know” stations. However, I detected explicit explanation only for natural selection. The question, “Why is genetic variation important?” is answered by saying “because it leads to natural selection, the mechanism that drives evolution.” *Where* that variation comes from, and the stochastic nature of that variation, is left unexplained. (A previous exhibit in the Field’s former evolution exhibit contained a roulette wheel and the provocative question, “Feel lucky, amphibian?” to convey the interactive effects of mutation and natural selection, but no such display is included in the current hall. See Asma 2001, p.204.)

### The Cleveland Museum of Natural History (Cleveland, Ohio); Coding, Genetic Drift, I

The museum’s small human evolution gallery focuses on fossil evidence, especially the earlier part of the hominin fossil record, consistent with the multi-decade association of the museum’s curators with Ethiopian paleoanthropology. An interactive game mentions sexual selection, “mutant” humans, and “radioactive mutant super-raccoons.” This same game includes hints of genetic drift in its suggestion that following a global catastrophe, a new human species could arise from a small, isolated population and, in an extraterrestrial option, the comment that “prolonged isolation is a powerful driver of evolution.” I certainly may have missed an explicit reference to natural selection, but all instances I noted referred simply to adaptation.

### The California Academy of Sciences (San Francisco, California); Coding: Natural Selection, E; Mutation, E; Genetic Drift, I

In *Human Odyssey*, natural selection is mentioned explicitly in connection with skin color. A colored lights display illustrates a population bottleneck and there is reference to a time (90–70 kya) when our species risked extinction. There is also a computerized migration display. The highly engaging Selam (infant *Au. afarensis*) diorama includes a computer station with progressive delivery of information. One section on species variation, in making a parallel to modern humans, explains that a large sample size is needed to appreciate our variability. While not specifically addressing forces/mechanisms, this component does promote statistical thinking.

An excellent exhibit just outside *Human Odyssey* on variation and natural selection contains wording similar to that at the Field Museum: “Variation fuels the process of evolution.” In the Naturalist area, one panel mentions interbreeding with Neanderthals. Mutation is mentioned explicitly in the context of eye evolution.

### The San Diego Museum of Man/The Museum of Us (San Diego, California); Coding: Natural Selection, E; Mutation, E

Mutation was covered in the *Footsteps Through Time* section, “Your DNA is unique.” Natural selection was explained along with adaptation to the environment in the same section that contained content on bacteria and antibiotic resistance. (A temporary exhibit in another museum building, *Cannibals: Myth and Reality*, included mention of mutation in the section on the PRNP gene; one variant allele provides protection from “a fatal brain disease” [i.e., kuru].) In the former human biology area, referred to by staff as the “Human Lab,” the “Choice or Chance” station allowed visitors to select traits or leave the outcome to genetic recombination. Additional “Human Lab” displays included gene selection and mtDNA. A series of human development exhibits in this space provided a parallel to the *Footsteps Through Time* evolution exhibits, but no explicit connection between evolution and development was made.

In the *Race* exhibit, both the migration and genetic variation animation and the one for skin color were out of order at the time of my data collection, but text explicitly refers to natural selection in the discussion of skin color. The concept of mutation, without the name, is included under the Q&A for genetic variation; mutation is mentioned directly in connection with sickle cell anemia and protection from malaria.

### The National Museum of Natural History, Smithsonian Institution (Washington, D.C.); Coding: Natural Selection, E; Mutation, E

The focus of the Smithsonian’s Hall of Human Origins is on the development of human “milestones” throughout the evolution of our species, with a strong environmental adaptation thread running through the hall’s exhibits. The hall includes genetic comparisons, indicating the close genetic similarity of all modern humans as well as our relationship to other species. In the FAQ computer interactive section, the answer to the question, “How Does Evolution Work?” does not explicitly mention mutation or natural selection but implicitly presents the VIST framework (see Box 1).

Elsewhere in the museum, remnants of an old Evolution Trail in the Hall of Mammals describe natural selection and adaptation. An Evolution at Work panel mentions mutation. A general evolution and paleontology section at the transition from the Ocean Hall to other halls contains text responding to the question, “Why do we need so many specimens?” with reference to variation: examining variation facilitates the identification of new species, and variation is the key to understanding the process of evolution. A museum exhibit on coevolution, “Partners in Evolution,” tells visitors that “evolution generates diversity” without description of the mechanisms.

### The American Museum of Natural History (New York, New York); Coding: Natural Selection, E; Mutation, E; Genetic Drift, E

The Hall of Human Origins at the AMNH was co-created by paleoanthropologist Ian Tattersall and molecular biologist Rob DeSalle in collaboration with an internal AMNH team. Due to DeSalle’s involvement, the coverage of the forces of evolution is explicit in the genetic section of the hall that parallels and complements its paleoanthropology partner. Variation is stated to be the source of all evolutionary change. The crucial question, “Where does variation come from?” is asked specifically. The answer includes genetic recombination as well as mutation. Recombination is critical to the generation of an enormous amount of genetic variation, so this explicit inclusion is significant. “Chance takes over” provides a clear and explicit explanation of genetic drift. In “recipe for a limb,” the concept of HOX genes is presented, without too many technical details. Other sections cover mtDNA and Y chromosomal inheritance as well as chromosome banding comparisons. An interactive computer station permits visitors to explore the Tree of Life. Regulatory genes are discussed in the context of changes in skeletal growth for *Homo ergaster* (*Homo erectus*, sensu lato) along with the Nariokotome subadult skeleton. Mutation is presented in the context of molecular clock estimates of dating along with migrations, but with the important caution that for dating, fossil evidence is superior.

Interestingly, this hall is not big on discussions of natural selection, especially as related to microevolution. Gene flow is also not emphasized. Skin color is connected to risk of skin cancer and vitamin metabolism, but evolution by natural selection is more explicitly discussed in connection with rapid brain evolution in recent human evolution and our distinction from previous hominins. The migration map section of the hall showing *Homo sapiens* migrations mentions that “countless later migrations mixed these early groups with one another,” but the hall does not highlight interbreeding. Instead, high species diversity is stressed. Nonhuman primate adaptations are described in the front portion of the hall, but adaptation is more often connected to the origination of distinct species. However, in the genetics portion of the first section of the hall, natural selection is mentioned near the display on recombination and mutation; while the latter are related to survival without mention of natural selection, a nearby display makes the point that which traits are advantageous depends on the environment.

### London’s Natural History Museum (London, England); Coding: Natural Selection, E; Gene Flow, I

As might be expected based on geographic location, Darwin pervades the Natural History Museum in London. However, explicit explanations of natural selection are rare. The pigeons display in the beautiful Treasures gallery does explicitly link artificial selection and natural selection in the historical context of the *Origin of Species*: “everyday pigeons provided crucial evidence for his theory that changed the world: evolution by natural selection.” Paleoanthropologist Chris Stringer discusses interbreeding among Neanderthals and *Homo sapiens* migrants in a video within the human evolution gallery; here, the interbreeding is connected to modern human adaptation to new environments and diseases. Genetic similarity with chimpanzees (ca. 98%) is quantified along with the comment that the similarity would be even higher with our extinct hominin relatives. While how natural selection operates is not explained, discussion of adaptation appears in several sections (e.g., bipedalism, ecological niches and new environments, and climate).

The Darwin Centre has as its main themes biodiversity and the importance of collections. Variation is discussed in the context of species: “If all the individuals within a species looked the same, we could easily tell they belong in the same group. But nature isn’t that simple. In any given species, individuals can vary – and this blurs the boundaries between groups. Sex, age and geographic distribution account for some of the differences.” Mutation is not explicitly discussed here. The Human Biology gallery contains sections on genetics, human development, and human reproduction. More could be done here to place human biology within an evolutionary framework.

## Discussion

The majority of the museums surveyed (10 of 12) explicitly cover natural selection within their exhibits (Table 1). Just over half (7 of 12) are similarly explicit in mentioning mutation. Only one, the American Museum of Natural History, explains genetic drift. The significance of gene flow in elevating variation within populations and thereby opposing the effects of genetic drift is not adequately emphasized in any of the exhibits.

There are several reasons museum exhibits might focus on natural selection with little description or discussion of other aspects of evolution. First, the VIST framework is fairly easy to explain to visitors because it builds on their understanding of natural selection and adaptation. Fewer visitors will have encountered descriptions of gene flow and genetic drift. More will likely have heard of mutation, the force/mechanism mentioned second-most commonly^3^.

Second, if the historical context of Darwin is introduced, focusing on natural selection and how Darwin deduced this mechanism is reasonable. This historical context, however, provides an opportunity to do more. Darwin’s inability to explain *how* new variations arose made his theory vulnerable to criticism. An effective response required the mid-20th-century theoretical developments of the Evolutionary Synthesis (see, e.g., Mayr and Provine 1998). Museum visitors could be guided through the same incremental understanding in an exhibit that presents the history of evolutionary theory. *Explore Evolution* comes close to doing this in its historical timeline^4^. Similarly, the Perot has a small section on key researchers and their contributions. However, such displays would be more effective if they situated museum visitors more directly in the shoes of the researchers who needed such advances in understanding to improve the credibility and explanatory power of evolutionary theory.

Third, natural selection may be easier to put on *visual* display. It is comparatively easy to show variations in the beaks of finches, for example, or fossil organisms that show changes in morphology over time. Visual content for mutation, genetic drift, and gene flow is more challenging. Nonetheless, as I argue below, we should try, and interactive computer technology can help.

### How to present more than natural selection and why that’s important

Museum exhibits have goals extending beyond the didactic. Indeed, as informal learning venues, they more often aim to ignite a spark and inspire curiosity (Thomas 2016). However, if visitors arrive at the museum without previous formal instruction in evolutionary theory, then exhibits that do not teach them the basic mechanisms of evolution can unintentionally contribute to erroneous conclusions. To use one example with practical consequences, some visitors to the Smithsonian’s Hall of Human Origins have concluded that we will adapt biologically to climate change. An interactive with simplistic adaptations, designed primarily for children, was thought to have contributed to this conclusion, so it was removed and replaced with a video on the Anthropocene (Briana Pobiner, personal communication). In part, such mistaken conclusions result from a lack of appreciation of Deep Time, but in part they derive from not understanding that natural selection operates on variation *already present* in *populations*. While it is unrealistic to teach museum visitors population genetics in one visit, providing some basic information and giving them an intuitive feel for the complexity of the interactions of evolutionary forces will, I submit, lessen mistaken prior assumptions. Exhibits that enhance visitor understanding of evolutionary forces need not be placed in human origins halls or fossil halls if achieving other seminal goals is primary, but this understanding is critical and merits space *somewhere* in science and natural history museums.

Box 2: summary of recommendations **Primary**

1. Include all forces (aka mechanisms) of evolution.
2. Explain the role of stochastic forces in evolution.
3. Explain the opposing effects on variation of genetic drift and gene flow.
4. Convey how complexity arises from simple components and processes and from the interaction of evolutionary forces over the span of Deep Time.
5. To accomplish goals 1–4, create a computer interactive experience or game that multiple users can engage with simultaneously.

**Secondary**

1. Consider using historical context to help visitors learn evolutionary principles.
2. Include more content for adult audiences; improve progressive delivery of information and scaffolding of ideas to enhance understanding of complex concepts.
3. Include more human examples to heighten interest in evolution (ancestry, evolutionary medicine).
4. Enhance content covering quantitative concepts.

This positive outcome is unlikely to occur due to textual content alone. (See Box 2 for a summary of my recommendations.) Most of us do not go to a museum to spend hours reading. We want to *see objects* and *experience things*. The desire for experiences motivates most modern museum goers (see, e.g., Pine and Gilmore 2019 and Walhimer 2022). Interactive museum technology has now advanced to the point that computerized simulations of the interactions of evolutionary forces could be designed. Such a presentation of simulations demonstrating the complex outcomes of interacting forces of evolution is an example of the effective use of technology in museums. People are unlikely to experience such a simulation outside a museum^5^ The simulation could be presented as a game with multiple players, further enhancing its appeal. For example, one player could control (i.e., enter values for) natural selection, another player could enter various values for gene flow and genetic drift (which have opposing effects on variation within and between populations), and a third could periodically introduce new mutations into populations of various sizes. Such a game would give visitors a much better intuitive understanding of how evolutionary forces operate than the exhibits currently on offer in museums.

One important issue is the level of information presented, corresponding to the educational attainment and targeted age of visitors. A game such as I suggest is comparatively sophisticated and unlikely to be of much value to children and young teens. Those with at least some high-school education are the targeted audience. Natural history and science museums should offer some proportion of their exhibits to these visitors. Good exhibits allow visitors to build on their knowledge over time, scaffolding new knowledge on top of what they have learned in previous museum visits or at younger ages. Older teenagers could enjoy playing such a game, and students learning about evolution in more formal academic settings would benefit greatly by interacting with one.

While no museum in my sample contains a computerized interactive activity for evolutionary forces, the Smithsonian’s “Keep Your Species Alive” interactive game, with up to three players working in concert or opposition simultaneously, served as an inspiration for the proposed simulation game suggested here. Another prototype is the natural selection game in Denver, which is effective in showing how trait frequencies can change in a population over time. Linking a game similar to this one with additional components for the other evolutionary forces would create the type of simulation I envision.

To best engage visitors, it might help to focus on human examples. (See Pobiner et al. 2018 for a study of A.P. biology high-school students indicating that a human focus is likely beneficial, at least for understanding natural selection.) For example, using skin color would be both interesting to visitors and would help dispel mistaken notions about the problematic concept of “race.” While many of my students have some previous knowledge of the relationship between skin color and ultraviolet radiation, that does not necessarily entail an understanding of why biological race is a problematic concept (see, e.g., Goodman et al. 2020). A simulation could include multiple mutations (including one for Neanderthals), show clinal variation along with the operation of gene flow subsequent to migration, genetic drift as related to population sizes, and the effects of natural selection through thousands of years in different environments. It could demonstrate that reproductive-age deaths (e.g., from skin cancer) make an evolutionary difference but that post-reproductive ones do not. Other simulations that explore evolutionary medicine would be similarly helpful and interesting to visitors. The relationship between sickle cell anemia and malaria is a classic example that also helps to counter simplistic concepts of race as related to medicine. Both skin color and sickle cell anemia are examples used in the *Race: Are We So Different?* exhibit.

### The importance of quantitative thinking

In advocating for presentation of more complex content on evolutionary mechanisms, let me highlight in conjunction the importance of preparing museum visitors and students for a sophisticated quantitative understanding of various facets of their natural and social worlds. An intuitive understanding of mathematics, statistics, and probability has value that extends beyond evolution. Consider risk assessment, epidemiological models of disease transmission, and the understanding of compound interest. People need to think quantitatively to understand how complex life can emerge from simple properties and principles. The computational biologist Andreas Wagner (2014) focuses on life’s ability to innovate. Understanding how molecules create phenotypes and how changing their interactions produces evolutionary change, he claims, is dependent on advances in mathematics. The significance of molecular interactions at the individual level has been similarly stressed by Lewontin (2000; see also Jamie Davies 2014). Petrosino et al. (2015), in arguing for “decentralized thinking” in teaching evolution, support the case I advocate here.

Understanding the manner in which so-called “random” factors can produce novel outcomes, some of which are beneficial, in both individual development and in evolution might mitigate the distaste many people have for the non-deterministic facets of change. I prefer to use the technical term “stochastic” to emphasize the unpredictability of mutation and genetic drift; this helps alleviate the concern over our existence being only due to chance. As defined by Oxford Languages Online (2022), “stochastic” means “randomly determined” or, more fully explained, “having a random probability distribution or pattern that may be analyzed *statistically* but may not be *predicted* precisely” (emphasis added). The focus here is on probability and prediction rather than lack of causation.

Yet another context in which an intuitive mathematical understanding is essential is in appreciating how cognition could evolve to the point of modern human conscious experience. This is, if not the greatest stumbling block to people’s acceptance of evolution, certainly among the chief reasons many are skeptical of evolutionary explanations for humans. (Think “irreducible complexity”^6^) In *Journey of the Mind*, Ogas and Gaddam (2022) build upon Stephen Grossberg’s hypothesis of the resonance of brain components and differential equation models to explain to readers how and why consciousness is more similar to the activity of a basketball game than to the computations of a computer. This is done conceptually, with no equations within the text of the book. Museums can similarly give visitors an intuitive feel for complexity and how it can evolve without the equivalent of mounting textbooks on display walls^7^.

## Conclusions

Two of twelve science and natural history museums in my sample did not include explicit explanations of any of the main forces/mechanisms of evolution (mutation, natural selection, genetic drift, and gene flow) in the exhibits surveyed. Three included explicit discussion only for natural selection, while six explained both mutation and natural selection. Only the American Museum of Natural History provided explicit discussion for three of these evolutionary forces. I argue that the development of computer interactive content will allow visitors to achieve an intuitive understanding of the complexity created by the interaction of evolutionary forces and will facilitate visitor comprehension of evolutionary mechanisms and evolutionary theory. A related benefit is enhancement of quantitative thinking.

## Data Availability

Data are not available. Data are qualitative (interviews, personal notes and photographs) and are being used for a more extensive monographic study.

## Footnotes

1 Spiegel et al. (2012) describe VIST as presenting evolutionary principles rather than evolutionary forces; this is an improvement in wording. While I use the common designation of “forces” throughout this article, I acknowledge the problematic nature of “force-talk” (Nehm et al. 2010).

2 I credit Nicole Burt, Curator of Human Health and Evolutionary Medicine, Cleveland Museum of Natural History, with emphasizing in our interview the need to “use the words” (personal communication, June 2017).

3 Even mutation, however, is used relatively infrequently in general public communications. Witness the common labeling of coronavirus “variants” without explaining how they arise and the need not to allow them free reign to increase in frequency due to selection for those proving most reproductively successful. (For an exception, see Doucleff 2021).

4 I consider *Explore Evolution* to be an excellent exhibit that accomplishes goals no other evolution exhibit I have seen does by placing human evolution in the broader context of the evolution of other organisms, from HIV to whales in scale and time, within a concentrated museum space. With due humility as a non-museum educator, I nonetheless encourage those who are museum educators to attempt to reach even more ambitious goals with their older visitors.

5 Simple simulations for selection, mutation, and drift are available at biologysimulations.com/evolution. More complex simulations are available to watch on the Primer site (youtube.com/c/PrimerLearning; the Simulating Natural Selection video has over 12 million views). A variety of simulations are used in classroom teaching. See Clarke-Midura et al. (2018) for an example. These require more time investment and/or instruction than is generally available in museums. In a museum environment, advantages would derive from interactivity and social facilitation, but the challenge would be maintaining interest. For a discussion of the game “Spore” with its developer Will Wright and evolutionary biologist Richard Prum, go to npr.org/transcripts/94563046; the issue of engagement vs. scientific accuracy arises in this conversation. Websites accessed 3 July 2022.

6 “Irreducible complexity” is a claim leveled against evolutionary theory by some creationists, associated with “intelligent design.” For one critique, see Young and Edis 2004.

7 If you doubt that such complicated exhibits can be done successfully, I invite you to consider the Canadian exhibit *Quantum*: https://themuseum.ca/exhibitions/past-exhibitions/quantum-the-exhibition/. Accessed 30 June 2022. On the creative possibilities of mutation and the quantum transitions between chemical states involved, see Carroll (2020).
